# Clinical effectiveness of alkasite versus nanofilled resin composite in the restoration of occlusal carious lesions in permanent molar teeth of children: a randomized clinical trial

**DOI:** 10.1007/s40368-023-00788-0

**Published:** 2023-03-22

**Authors:** H. Sharma, B. S. Suprabha, R. Shenoy, A. Rao, H. Kotian

**Affiliations:** 1 Department of Pediatric and Preventive Dentistry, Manipal College of Dental Sciences, Mangalore, Manipal Academy of Higher Education, Light House Hill Road, Mangalore, Karnataka 575001 India; 2 Department of Pediatric and Preventive Dentistry, Manipal College of Dental Sciences, Mangalore, Manipal Academy of Higher Education, Manipal, India; 3 Department of Public Health Dentistry, Manipal College of Dental Sciences Mangalore, Manipal Academy of Higher Education, Manipal, India; 4 Department of Community Medicine, Kasturba Medical College Mangalore, Manipal Academy of Higher Education, Manipal, India

**Keywords:** Nanocomposite, Alkasite, Molar, Dental restoration, Permanent

## Abstract

**Purpose:**

To evaluate and compare the clinical effectiveness of alkasite with nanofilled resin composite restorations for occlusal caries lesions in permanent molar teeth of children, at one-year follow-up.

**Methods:**

In this randomized controlled clinical trial with parallel design, 38 children aged 7–13 years with occlusal caries lesions on 59 first permanent molars were randomly allocated into two groups, Group 1: Filtek™ Z350XT (nanocomposite) and Group 2: Cention N^®^ (alkasite resin composite). The restorations were evaluated at one year using the United States Public Health Service (USPHS) criteria. Data were analyzed using Chi-square or Fisher’s exact test.

**Results:**

All restorations had either Alpha or Bravo scores at one-year follow-up. In Group 1, all restorations scored Alpha, while one restoration each (3.6%) in Group 2 scored Bravo for fracture and marginal adaptation. All restorations in both groups scored Alpha for retention, secondary caries, and post-operative sensitivity. For anatomic form, all restorations in Group 1 scored Alpha, while three (10.7%) restorations in Group 2 had Bravo scores. For marginal discolouration, three restorations in both groups scored Bravo (11.5% and 10.7%, respectively). For surface roughness, one restoration (3.8%) in Group 1 and three restorations in Group 2 (10.7%) scored Bravo. The comparative results between the two groups for all the variables in the USPHS criteria were not statistically significantly different.

**Conclusions:**

The performances of the nanofilled composite and alkasite were clinically acceptable and comparable. Alkasite can be an alternative material for the restoration of occlusal caries lesions in permanent molars of children.

**Clinical trial registration:**

The clinical trial was registered at Clinical Trials Registry—India (CTRI Reg no: CTRI/2020/12/029830 Dated: 15/12/2020).

## Introduction

Dental resin composites have gradually replaced amalgam as the standard restorative material for permanent teeth in following the principles of minimally invasive dentistry and the treaty of the Minamata Convention on Mercury in 2013 that encourages phase down of amalgam (Celik et al. 2010; Fisher et al. 2018). Nevertheless, they are known to have drawbacks such as inadequate resistance to wear, marginal leakage and occurrence of secondary caries (Sadeghi et al. 2010). The development of nanofilled composites containing strontium fluoro-aluminate silicate glass particles with an average particle size of 1–100 nm improved the properties significantly and are employed for restoration in high stress-bearing areas (Celik et al. 2010; Alzraikat et al. 2018). Nanoparticles form loosely bound agglomerates that help in the reduction of the interstitial spacing between filler particles and therefore, contribute to increased filler loading, improved physical properties, and polish retention (Bastos et al. 2021). Resin composites have a poor prognosis when compared to amalgam restorations due to polymerization shrinkage, inferior strength, and failure of the bond between fillers and resin. Additionally, the water solubility of the silane-coupling agents affects water sorption and wear resistance of the material leading to frequent repairs. (Soncini et al. 2007; Alzraikat et al. 2018).

Recently, an alkasite-based tooth-coloured material, Cention N^®^ has been introduced as an alternative to amalgam for clinicians. Alkasite is a resin-based bioactive bulk fill restorative material (Todd 2016; Ilie 2018). It is a subgroup of the resin composite material class containing alkaline fillers capable of releasing acid-neutralizing hydroxyl ions, which regulate the pH during acid attacks, preventing demineralization. Moreover, the release of fluoride and calcium ions leads to the remineralisation of dental enamel (Todd 2016; Tiskaya et al. 2019).

In-vitro studies conducted in the recent past with alkasite resin composite show that alkasite has better or similar mechanical and biological properties such as microleakage, microhardness, fracture resistance and bond strength, to those of other resin composites (George and Bhandary 2018; Mazumdar et al. 2018; Jayashankara et al. 2020; Awad et al. 2020). Only a few in-vivo studies have been conducted assessing the performance of the alkasite when used for restorations in permanent molars and all involved adult dental patients. An in vivo study conducted assessing the clinical performance of Cention N^®^ in class I cavities concluded that it is a suitable replacement for amalgam, in the restoration of posterior teeth (Dedania et al. 2018). However, another in-vivo study that compared the post-operative sensitivity in three types of bulk fill materials used for the restoration of class I cavities concluded that Cention N^®^ resulted in more post-operative sensitivity than other bioactive materials like glass hybrid restorative material or reinforced resin-modified glass ionomer, at one-month follow-up (Hirani et al. 2018). No in-vivo study is available in the literature to compare the clinical effectiveness of this alkasite as a permanent molar restorative material with commonly used resin composites such as nanocomposites.

Therefore, the aim of the current study was to evaluate the clinical effectiveness of an alkasite with nanofilled resin composite in the restoration of occlusal carious lesions in permanent molar teeth of children. The null hypothesis was that there is no difference between the clinical effectiveness of alkasite resin composite and nanofilled resin composite as a permanent restorative material for occlusal carious lesions in permanent molar teeth of children.

## Material and methods

This clinical study had a randomised controlled, two-arm parallel design and is reported according to the Consolidated Standards of Reporting Clinical Trials (CONSORT) guidelines and is registered in the database for the registration of clinical studies (Clinical Trial registry of India, Reg. No.: CTRI/2020/12/029830). The participants selected for this study were outpatients of the Department of Paediatric and Preventive Dentistry.

### Compliance with ethical Standards

The Institutional Ethics Committee approved the research protocol (Protocol Reference: 19084). Parents of the participants were informed about the procedure using a patient information sheet and written informed consent was obtained by the principal investigator. Written informed assent from the participating children was also obtained. Participation or non-participation in the study did not interfere with their dental treatment and no compensation was provided to the participants.

### Sample size

The sample size calculation was performed at a 90% confidence interval and power of 80%, assuming the effect size to be 25% and a failure rate (fracture of restoration) of 3% (Loguerico et al. 2019). The total sample size of the study was calculated to be 26. Assuming a loss to follow-up of 10%, the final sample size for the study was 29 in each group. The calculation of sample size was done using the website www.sealedenvelope.com, available online for free (Sealed Envelope 2012).

### Eligibility criteria

Children aged 7–13 years, with carious lesions on the occlusal surface in any one of the first permanent molars with ICDAS score ranging from 3–5 (Ismail et al. 2007) were selected for the study. To be included in the study, the children were also required to have satisfactory oral hygiene with a simplified oral hygiene index score ≤ 3 (Green and Vermillion 1964) and Frankl’s Behaviour rating scale score of 3 or 4 (Stigers 2016). Additionally, the first permanent molars should have had their occlusal surfaces fully visible with no operculum covering the occlusal surface and the gingival tissue below the level of the distal marginal ridge.

The exclusion criteria were as follows: permanent molars with deep dentinal caries or presence of any pulpal disease with or without pain, permanent molars with developmental defects such as hypomineralisation or hypoplasia, participants with a history of allergy to any material being used in the study, a history of abnormal parafunctional activity, and participants with mental disabilities and systemic illness.

### Randomisation

Permuted block randomisation with block sizes 4 and 8 was used. Blocks were randomly allocated using a random table of numbers. An operator who was otherwise not participating in the study assigned participants to one of the groups. The allocation ratio was 1:1. The group to be assigned was marked on cards enclosed in sequentially numbered, opaque, sealed envelopes. After the cavity preparation and before the commencement of the restorative treatment, the assigned group was disclosed to the lead investigator who conducted the restorative treatment. Each participant was a unit of randomisation. For a given participant, all eligible permanent molars were restored with the same restorative material, as per group allocation as follows:

Group 1: Nanofilled resin composite material Filtek™ Z350XT (3 M ESPE, St. Paul, MN, USA, A2 shade).

Group 2: Alkasite resin composite material Cention N^®^ (Ivoclar Vivadent Inc, NY, USA, A2 shade).

The composition of the materials used for the study is given in Table 1.

**Table 1:** Composition of principal materials used in the study

Material	Composition	Manufacturer and lot number
Cention N^®^	Filler: Barrium–aluminium silicate glass (20–30%) Calcium fluorosilicate glass (25–35%), Isofiller (15–25%) Ytterbium fluoride (5–10%) Monomer: Di-methacrylate (95–97%) Stabilizer (< 1%), Initiator (< 1%), Pigment (< 0.1%), Additive (1–2%)	Ivoclar Vivadent Inc, NY, USA. Lot Number: N601734
Filtek™ Z350	Organic phase: UDMA, BisGMA, BisEMA, TEGDMA Inorganic matrix: Silica (20 nm non-agglomerated and/or aggregated), zirconia (4–11 nm non-agglomerated and/or aggregated and agglomerated), clusters (size of the cluster ranging from 0.6 to 1.4 μm, 59.5 in volume percentage), zirconia/silica aggregated particles (20 nm particles of silica along-with with 4–11 nm sized zirconia**)**	3M ESPE, St. Paul, Minnesota, USA. A2 shade. Lot Number: 684196AN

### Clinical procedure

All the restorative procedures were done under rubber dam isolation by a single operator (Principal Investigator). The included molars were cleaned, polished with non-fluoridated prophylaxis paste, rubber cup and micromotor handpiece to remove any plaque and debris. A topical anaesthetic (10% lidocaine spray) (Neon Laboratories Limited, Mumbai, India) was applied before rubber dam isolation (Hygenic^®^Dental Dam 5 × 5inches, Coltène/Whaledent INC., Altstätten, Switzerland). A local anaesthetic [LOX 2% lignocaine with adrenaline (1:200,000)] (Neon Laboratories Limited, Mumbai, India) was administered for caries extending to dentin (ICDAS score 4 and 5).

### Cavity preparation

Carious tissue was removed using a conservative cavity preparation method. The outline form of the cavity was defined by the extent of the carious lesion. Unsupported enamel (enamel unsupported by dentin) was preserved, while undermined enamel (thin or loose enamel) margins were removed. Extensions towards the marginal ridge retained at least 2 mm of the remaining tooth surface (Manhart et al. 2008). The preparation was done using an SF-31 ISO 109/013 straight flat-ended diamond-coated bur (MANI INC., Shioya, Japan) for initial access and caries on the periphery of the cavity was removed using a BR-31 ISO 001/018 ball round type diamond-coated bur (MANI INC., Shioya, Japan), using high-speed hand piece (NSK America Corporation, Hoffman Estates, Illinois, USA) under air–water spray. Remaining caries on the pulpal floor were removed using a spoon excavator until firm dentin was reached (Innes et al. 2016). The caries removal to firm dentin was confirmed by visual and tactile method (with a mouth mirror and ball-ended probe). No liner was applied before the restoration. No bevelling was done at the enamel cavosurface. All restorations were performed according to the instructions of the manufacturer.

#### Etching and bonding

Group 1: The tooth surface was etched with 3M ESPE Scotch-bond Etchant (37% phosphoric acid gel) (3M ESPE, St. Paul, MN, USA) with an applicator brush for 15 s. The tooth surface was washed with a three-way syringe for 10 s and gently dried by blotting with a mini sponge to give a moist, glistening appearance, which ensured that the tooth structure was not overdried. Using an applicator brush, two even coats of etch-and-rinse dental adhesive (3M single bond 2 adhesive) (3M ESPE, St. Paul, MN, USA) were applied to enamel and dentin, gently agitated for 20 s to facilitate better penetration of the adhesive into the dentinal structure, followed by drying gently for a period of 2–5 s to evaporate the solvent and then light curing for 10 s.

Group 2: Alkasite was bonded after selective etching of enamel. 3M ESPE Scotch-bond Etchant (37% phosphoric acid gel) (3M ESPE, St. Paul, MN, USA) was applied with an applicator brush only on the enamel for a period of 15 s. The tooth surface was then washed with a three-way syringe for 10 s and gently dried to give a moist, glistening appearance. Tetric N-Bond Universal (Ivoclar Vivadent Inc, New York, USA), a multimode dental adhesive, was applied to the surface of both enamel and dentin using an applicator brush, agitated for 20 s, dried with compressed air for 2 s to evaporate the solvent, followed by light curing for 10 s.

#### Placement of restorative material

For both groups, the material was placed into the cavity with help of a plastic filling instrument, followed by condensation with a composite condenser.

Group 1: Composite material was placed in split horizontal increments of 1.5 mm.

Group 2: Composite material was placed as a single increment. The powder and liquid of the alkasite composite were mixed as per the instructions of the manufacturer until a homogeneous consistency was obtained and condensed within 3-min working time.

#### Curing time

LED light curing device (Bluedent, BG Light Ltd, Plovdiv, Bulgaria) was used at a constant intensity of ≥ 500 mW/cm^2^. Light curing was performed for 20 s and 40 s in Groups 1 and 2, respectively. Immediately after curing the restoration, occlusion was checked with an articulating paper. Finishing and polishing were done with a contra-angle micromotor (NSK America Corporation, Hoffman Estates, Illinois, USA) at a slow speed with a constant flow of water for cooling. Finishing of the restorations was performed using a TR-26EF ISO 195/018 FG extra fine composite polishing bur (MANI INC., Shioya, Japan). Polishing of the restorative materials was done with Enhance Polishing System (Denstply Sirona, York, PA, USA). All restorations were cross-checked for quality and adequateness by an experienced examiner before the patients were sent away from the dental operatory.

### Evaluation

After one year, the restorations were evaluated by two examiners who were previously trained and calibrated, using Modified Ryge/USPHS (United States Public Health Service) criteria (Bayne and Schmalz 2005; Canali et al. 2019). During the follow-up examination, the restored teeth were examined under artificial light after drying, using a mouth mirror and ball-ended probe. The sensitivity of the restorations was assessed by blowing a stream of compressed air and tactile contact with the probe. A history of sensitivity to cold or hot food substances was obtained from the participants. Any marginal discoloration indicative of caries, with ditching or cavitation along the margins of the restoration observed during the visual and tactile examination, was considered as secondary caries. Alpha and Bravo score meant an acceptable clinical result; while a Charlie and Delta score referred to an unacceptable restoration that required a replacement or repair of the existing damage (Canali et al. 2019).

#### Calibration of examiners

Both the examiners were trained and calibrated for the USPHS criteria before the evaluation of the restorations (Table 2). Intra-examiner and inter-examiner reliability was calculated from the scores of ten randomly selected participants during the study. Intra-examiner reliability was checked for the two examiners at baseline and one-week intervals and a Cohen’s Kappa value = 1 was obtained for both examiners, indicating total agreement. Inter-examiner reliability Kappa values ranged from 0.67 to 1, depicting moderate agreement.

**Table 2:** Description of the criteria for clinical evaluation of the restorations at follow-up using the USPHS criteria (modified from Canali et al. 2019)

Category	Criteria			
	Rating	Acceptable	Rating	Un-acceptable
Fracture	Alpha	Absent	Charlie	Clinically unacceptable fracture
	Bravo	Small fracture, clinically acceptable		
Anatomic form	Alpha	The restoration is continuous with tooth anatomy	Charlie	Restoration is under-contoured, dentin or base exposed
	Bravo	Slightly under- or over-contoured restoration	Delta	Restoration is missing or partially fractured; restoration causes pain in tooth or adjacent tissue
Secondary caries	Alpha	No evidence of caries contiguous with the margin of the restoration	Charlie	Caries is evident contiguous with the margin of the restoration
Marginal discoloration	Alpha	No discoloration evident	Charlie	Obvious staining cannot be polished away
	Bravo	Slight staining, can be polished away	Delta	Gross staining
Marginal adaptation/ integrity	Alpha	Restoration is contiguous with existing anatomic form, explorer does not catch	Charlie	Crevice at margin, enamel exposed
	Bravo	Explorer catches, no crevice is visible into which explorer will penetrate	Delta	Obvious crevice at margin, dentin or base exposed
Post-operative sensitivity	Alpha	No postoperative sensitivity, after the restorative procedure and during the study	Charlie	Sensitivity at any stage of the study
Surface texture/roughness	Alpha	Smooth surface	Charlie	Rough, cannot be polished
	Bravo	Slightly rough or pitted, can be polished	Delta	Surface deeply pitted, irregular grooves
Retention	Alpha	No loss of restorative material	Charlie	Missing restoration
	Bravo	Partial loss of restorative material		

#### Blinding

The study participants, the examiners who performed the follow-up examination and the statistician who carried out the analysis were blinded to group allocation. However, the operator who had carried out the restorative procedures was not blinded to the group allocation.

### Statistical analysis

Data were entered and analyzed using IBM SPSS Statistics for Windows, version 20 (IBM, Armonk, NY, USA). Descriptive statistics were tabulated. The baseline data and the outcomes were compared between the groups using the Students *t* test, Chi-square test and Fisher’s Exact test. The statistical analysis was done according to the intention to treat principle. The statistical significance was set at *p* ≤ 0.1.

## Results

**Fig. 1:**
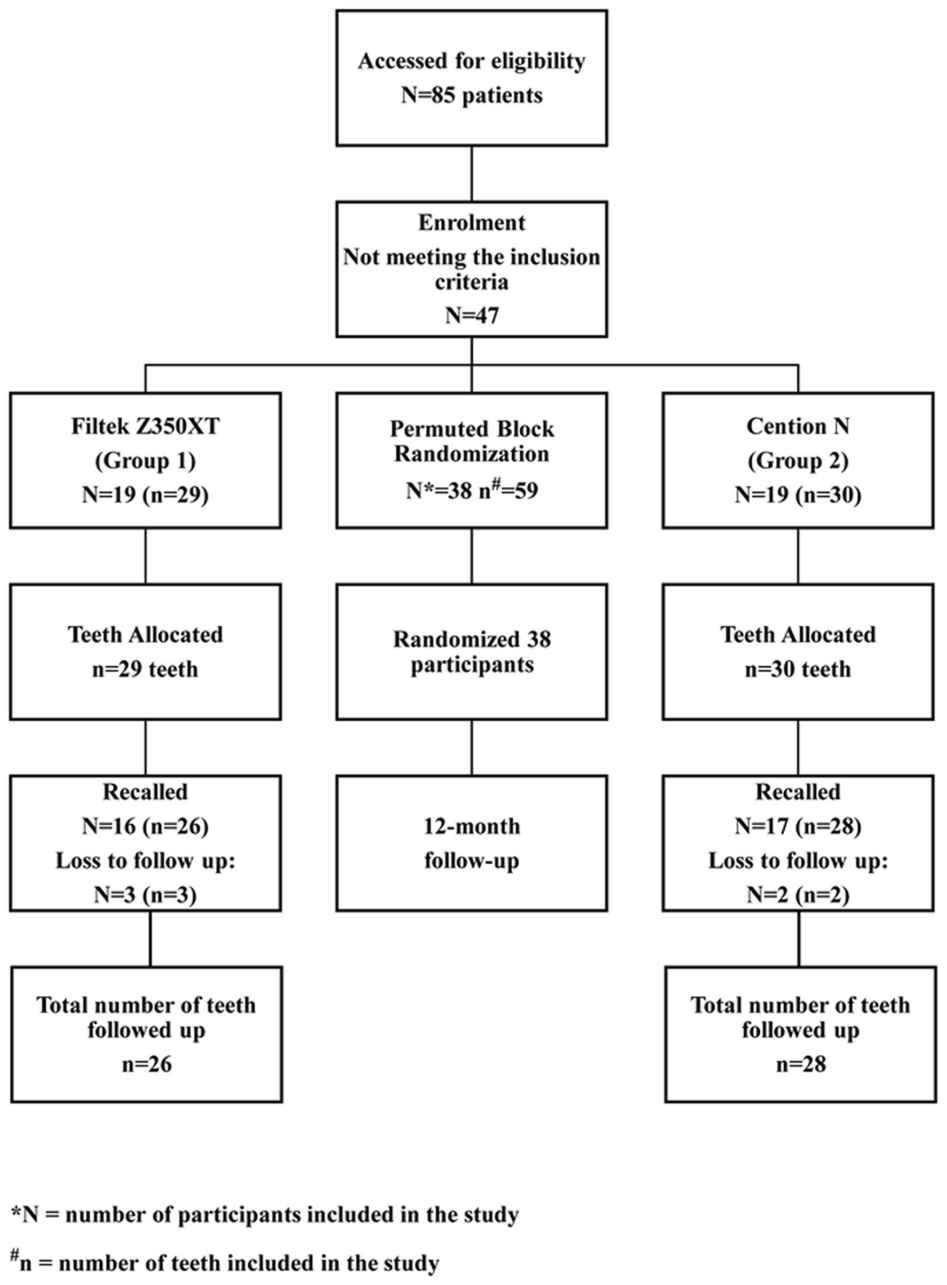
Flow diagram representing flow of participants until 12 months in the study

At the beginning of the study, 38 children with 59 erupted first permanent molars were recruited from December 2020 to February 2021(Fig. 1). The mean age of the selected participants for the study was 10.32 ± 1.80 years (Table 3). The final sample consisted of 17 males and 42 females. The chi-square test showed no significant difference in gender between the groups (Table 4). The treatment period was from December 2020 to March 2021. Comparison of baseline characteristics between the groups such as OHIS, DMFT and dmft, ICDAS scores revealed no significant difference statistically. (Tables 3 and 4) Most children in the sample had good oral hygiene (86.2% and 73.3% for Group 1 and Group 2, respectively). The frequency of ICDAS score 3 was higher in both Group 1 and Group 2 (58.6% and 63.3%, respectively). Overall, the number of mandibular molars (around 60%) was greater than that of maxillary molars (around 40%) in the sample. No statistically significant difference was observed between the groups based on the type of molar (maxillary/mandibular, right/left) included in each group and the number of molars included per participant. Most of the participants in both groups had only one molar included in the study (57.9% and 52.6% in Group 1 and 2, respectively) (Table 4).

**Table 3:** Comparison of Mean Age, Baseline Mean DMFT and dmft scores between the groups

Variable	Filtek™ Z350 XT (nanofilled resin composite) mean ± S.D	Cention N^®^ (alkasite resin composite) mean ± S.D	*t* value	*p* value
Age	10.76 ± 1.81	9.90 ± 1.71	1.88	0.066
DMFT Score	1.83 ± 0.76	1.87 ± 0.73	0.20	0.841
dmft Score	0.45 ± 1.02	0.60 ± 1.19	0.52	0.602

**Table 4:** Comparison of frequencies of various characteristics of the groups at baseline

Characteristics		Filtek™ Z350 XT (nanofilled resin composite) (%)	Cention N^®^ (Alkasite resin composite) (%)	Chi-square	*p* value
Sex	Male	7 (24.1)	10 (33.3)	0.61	0.436
	Female	22 (75.9)	20 (66.7)		
OHIS index	Good	25 (86.2)	22 (73.3)	1.51	0.219
	Fair	4 (13.8)	8 (26.7)		
ICDAS score at baseline	Score 3	17 (58.6)	19 (63.3)	–	0.892*
	Score 4	11 (37.9)	11 (36.7)		
	Score 5	1 (3.4)	0 (0.0)		
Type of molar included	Tooth #16	7 (24.1)	5 (16.7)	3.12	0.373
	Tooth #26	8 (27.6)	4 (13.3)		
	Tooth #36	8 (27.6)	11 (36.7)		
	Tooth #46	6 (20.7)	10 (33.3)		
Number of molars included per participant	1	11 (57.9)	10 (52.6)	–	0.940*
	2	6 (31.6)	7 (36.8)		
	3	2 (10.5)	2 (10.5)		

*Fisher’s Exact test

The follow-up period lasted from December 2021 to March 2022. After a one-year follow-up, 33 participants (54 molars) were evaluated using USPHS criteria. Five participants (number of molars = 5) did not report on the days of examination and were untraceable due to migration to other cities during the follow-up period. The results are shown in Table 5. In Group 1, all restorations had an Alpha score, with none of the restorations showing fracture (100.0%). In Group 2, all restorations scored Alpha (96.4%) except one restoration that showed a small fracture that was clinically acceptable and hence got a Bravo score (3.6%). No statistically significant difference was observed between the two groups for the fracture outcome. In terms of marginal discolouration, three restorations in both Group 1 (11.5%) and Group 2 (10.7%) had a Bravo score (slight staining that can be polished away). In Group 1, all restorations showed an Alpha score with no loss of marginal adaptation (100.0%). However, for Group 2, all restorations scored Alpha except one that had a Bravo score (3.6%), with a catch at the margin on probing and no visible crevice. In Group 1, all restorations showed an Alpha score except one with a Bravo score (slightly under/over contoured restoration) (3.8%) for anatomic form criteria, while in Group 2, three restorations had a Bravo score (10.7%). For surface roughness criteria, in Group 1, one restoration and in Group 2, three restorations showed a Bravo score (slightly rough or pitted that can be polished) (3.8% and 10.7%, respectively). There were no significant differences in the marginal discolouration, marginal adaptation, anatomic form, and surface roughness criteria between the groups. None of the restorations in both groups showed loss of retention, post-operative sensitivity, or secondary caries. Since all restorations had either Alpha or Bravo scores at follow-up, the performances of both restorative materials were deemed clinically acceptable.

**Table 5:** Number (percentage) of evaluated restorations for each experimental group, classified according to the modified USPHS criteria

Evaluated criteria	Score	Filtek™ Z350 XT (nanofilled resin composite) (%)	Cention N^®^ (alkasite resin composite) (%)	*p* value
Fracture	Alpha	26 (100.0)	27 (96.4)	1.000*
	Bravo	0 (0.0)	1 (3.6)	
	Charlie	0 (0.0)	0 (0.0)	
Anatomic form	Alpha	26 (100)	25 (89.3)	0.237*
	Bravo	0 (0.0)	3 (10.7)	
	Charlie	0 (0.0)	0 (0.0)	
	Delta	0 (0.0)	0 (0.0)	
Secondary caries	Alpha	26(100.0)	28(100.0)	0
	Charlie	0 (0.0)	0 (0.0)	
Marginal discolouration	Alpha	23 (88.5)	25 (89.3)	1.000*
	Bravo	3 (11.5)	3 (10.7)	
	Charlie	0 (0.0)	0 (0.0)	
	Delta	0 (0.0)	0 (0.0)	
Marginal adaptation/integrity	Alpha	26 (100.0)	27 (96.4)	1.000*
	Bravo	0 (0.0)	1 (3.6)	
	Charlie	0 (0.0)	0 (0.0)	
Post-operative sensitivity	Alpha	26 (100.0)	28 (100.0)	0
	Charlie	0 (0.0)	0 (0.0)	
Surface texture/roughness	Alpha	25 (96.2)	25 (89.3)	0.612*
	Bravo	1 (3.8)	3 (10.7)	
	Charlie	0 (0.0)	0 (0.0)	
	Delta	0 (0.0)	0 (0.0)	
Retention	Alpha	26 (100.0)	28 (100.0)	0
	Bravo	0 (0.0)	0 (0.0)	
	Charlie	0 (0.0)	0(0.0)	

*Fisher’s Exact test

## Discussion

In this study, two different classes of resin composites were utilised for the restoration of occlusal surfaces of the first permanent molars in paediatric dental patients. The clinical effectiveness of a nanofill (Filtek™ Z350XT) resin composite was compared with an alkasite (Cention N^®^) composite at a one-year follow-up. Based on the results, the null hypothesis was accepted.

Despite both groups demonstrating clinically acceptable outcomes, some results were noteworthy in both groups. In Group 1 (nanocomposite), Bravo scores were obtained in three restorations for marginal discolouration and one restoration for surface texture at the end of the evaluation. In Group 2 (alkasite), three restorations scored Bravo for anatomic form, surface roughness and marginal discolouration each, while one restoration each had a Bravo score for marginal adaptation and fracture at the end of the evaluation.

A fracture is a complete or incomplete break in the restorative material, during the application of force and has been the primary cause of failure of resin composite restorations (Jayashankara et al. 2020). Only one restoration in the Cention N^®^ group showed a fracture that was clinically acceptable and repairable, which is in accordance with earlier in-vitro studies on fracture resistance that showed Cention N^®^ has better or comparable resistance to fracture as nanocomposite or nanohybrid composite (Jayashankara et al 2020; Firouzmandi et al. 2021).

Both materials showed clinically acceptable levels of marginal discolouration and marginal integrity in all restorations, which is explained by their low polymerisation shrinkage properties. Marginal adaptation of the material depends on forces produced due to polymerisation shrinkage that causes debonding of the material at the tooth interface, often leading to discolouration at the margins (Yazici et al. 2017). Filtek™ Z350 XT, a nanofilled resin composite, has a resin system that has lower double bonds per unit weight, leading to lower shrinkage (Askikfgajer et al. 2002). Cention N^®^ is an alkasite dual cure resin composite that is UDMA based and contains iso-fillers that lower the stress, leading to low shrinkage (Ilie 2018). The split incremental technique used for the restoration in the nanocomposite group reduced the C factor, which is the highest in Class I restorations, leading to decreased shrinkage of the nanocomposite (Chandrasekhar et al. 2017). However, the bulk filling resin composites like alkasites also show decreased polymerisation shrinkage due to the presence of isofillers which are prepolymerized filler particles functionalized with silane, high flexural strength, and low modulus of elasticity (Todd 2016, Ilie 2018).

Similar results for nanofilled composite have been reported in a study, where most of the nanocomposite restorations received Alpha and Bravo scores for marginal adaptation after a 30-month follow-up (de Andrade et al. 2011). Another study also reported only superficial marginal staining of nanofilled composite restorations at 6-month evaluation (Frascino et al. 2020). The results for marginal adaptation in this study for the alkasite group were also similar to an earlier clinical study, where all the Cention N^®^ restorations showed Alpha scores for marginal adaptation at six-month and one-year follow-up (Dedania et al.^2018^).

Loss of marginal adaptation can also occur due to localised bond failure. While etching with phosphoric acid increases the bond strength to enamel, it decreases the bond strength to dentin leading to adhesion failures. Hence, it is recommended that only enamel should be selectively etched (van Landuyt et al. 2006), which was performed in this study in Group 2. In addition to forming resin tags and a hybrid layer, the adhesive also chemically bonds with the calcium molecules in the hydroxyapatite because of 10-MDP (10-Methacryloyloxydecyl dihydrogen phosphate) functional monomer present in it. Use of adhesive also compensates for the C-factor, with the adhesive layer acting as an intermediary stress reliever (Yao et al. 2020). In the case of the nanocomposite, where the adhesion is based on the total-etch concept, a hybrid layer is formed that consists of hybridised dentinal tubules and resin tags. This hybrid layer extends to the maximum depth of demineralised dentin due to acid etching, thus achieving a close adaptation of the material with both dentin and enamel (Hegde et al. 2020). The excellent bonding between the nanocomposite and the tooth structure may have resulted in good marginal adaptation results of Group 1 in this study.

Loss of retention was not reported in both groups, which is explained by the bond strength of both the restorative materials and their mechanical properties such as wear resistance, modulus of elasticity and flexural strength reported in earlier studies on nanocomposite and alkasite (Alzraikat et al. 2018; Chole et al. 2018; Roulet et al. 2020; Naz et al. 2021). The mechanical properties of Cention N^®^ have been credited to the presence of barium–aluminium–silicate and calcium–aluminum–silicate glass-based filler particles and cross-linked structure of the polymer (Todd 2016).

Filtek™ Z350 nanocomposite has higher filler loading and smaller-sized filler particles, enabling a reduction in the interstitial spaces. This preserves the soft resin matrix and lowers filler particle loss on the surfaces, improving wear resistance and maintaining the smooth surface texture of the restoration (de Paula et al. 2011). This explains the surface texture of the nanocomposite restorations at follow-up with only one tooth showing a Bravo score for surface roughness. Alkasites have a lower filler load than nanocomposites; the fillers are larger and irregular, leaving voids after polishing and hence higher surface roughness. An earlier in-vitro study has noted higher surface roughness of Cention N^®^ compared to nanocomposite, especially when subjected to chewing simulation (Naz et al. 2021). Both the alkasite and nanocomposite materials have high wear resistance, that is comparable to nanohybrid and bulk-fill composites (Alzraikat et al. 2018; Naz et al. 2021), which explains the clinically acceptable level performance in terms of anatomic form in both the groups.

The results of our research showed that the clinical effectiveness of Cention N^®^ is similar to that of nanocomposite at one-year follow-up. However, contrary to the results of our study, higher post-operative sensitivity has been reported for Cention N^®^, which could be due to the placement of Cention N^®^ without adhesive in a conventionally prepared cavity (Hirani et al. 2018). In our study, Cention N^®^ was used with a universal adhesive after conservative cavity preparation.

Based on the literature search done for this study, this randomised controlled trial with nanocomposite as the positive control group is the first of its kind conducted among paediatric dental patients. Composite resin restoration on a permanent first molar in children has more limitations than in adult patients, due to its proximity to the gingiva and lack of patient compliance, both of which affect isolation (Donly and Garcia-Godoy 2015). The bonding of resin composites to permanent teeth in children may be influenced by the morphology and chemical composition of enamel and dentin which is different than that of older individuals, possibly leading to different clinical outcomes for resin composite restorations in children (Sheen et al. 1993; Gjorgievska et al. 2008). The results of this study are applicable towards choosing a posterior restorative material for treating occlusal surface caries in permanent teeth of children with good oral hygiene and showing cooperative behaviour during dental treatment. Cention N^®^ has the advantage of being tooth-colored material with fluoride and hydroxyl ion-releasing properties, improved handling, and mechanical properties at a reduced cost (Todd 2016), giving it a promising scope as a restorative material.

The limitations of the study include a short follow-up period and an assessment of post-operative sensitivity subjectively based on the participant’s response. The study used a parallel design that results in inter-participant variability, thus introducing selection bias (Lesaffre et al. 2009). Though well-defined inclusion criteria minimized variation among the participants, mastication forces, occlusal habits, temperature, abrasive, and chemical properties of foods consumed, salivary properties of the individual participant are uncontrollable factors that affect composite restoration’s longevity (Sarrett 2005). All restorations were placed by a single operator who was not blinded, leading to performance bias, but was minimized due to close supervision during the placement of the restorative materials. Further research with a long-term follow period is required to understand the prognosis of the alkasite restorations in comparison with other resin composite materials. Future studies can also consider cavity extension (size and depth) while evaluating the survival of alkasite restorations.

## Conclusion

The clinical effectiveness of Filtek™ Z350XT (nanocomposite) and Cention N^®^ (alkasite) used for the restoration of occlusal caries lesion in the first permanent molars of children did not differ significantly at one-year follow-up, with both showing acceptable clinical performance. Both Cention N^®^ (alkasite) with adhesive and Filtek™ Z350 XT (nanocomposite) can be used for the restorations of occlusal cavities in permanent posterior teeth of children. Considering the study limitations, further long-term clinical trials are needed for more decisive inferences.

## Data Availability

The data that support the findings of this study are available from the corresponding author upon reasonable request.
